# Unveiling Interactions among Mitochondria, Caspase-Like Proteases, and the Actin Cytoskeleton during Plant Programmed Cell Death (PCD)

**DOI:** 10.1371/journal.pone.0057110

**Published:** 2013-03-06

**Authors:** Christina E. N. Lord, Adrian N. Dauphinee, Rebecca L. Watts, Arunika H. L. A. N. Gunawardena

**Affiliations:** Department of Biology, Dalhousie University, Halifax, Nova Scotia, Canada; University College Dublin, Ireland

## Abstract

*Aponogeton madagascariensis* produces perforations over its leaf surface via programmed cell death (PCD). PCD begins between longitudinal and transverse veins at the center of spaces regarded as areoles, and continues outward, stopping several cells from these veins. The gradient of PCD that exists within a single areole of leaves in an early stage of development was used as a model to investigate cellular dynamics during PCD. Mitochondria have interactions with a family of proteases known as caspases, and the actin cytoskeleton during metazoan PCD; less is known regarding these interactions during plant PCD. This study employed the actin stain Alexa Fluor 488 phalloidin, the actin depolymerizer Latrunculin B (Lat B), a synthetic caspase peptide substrate and corresponding specific inhibitors, as well as the mitochondrial pore inhibitor cyclosporine A (CsA) to analyze the role of these cellular constituents during PCD. Results depicted that YVADase (caspase-1) activity is higher during the very early stages of perforation formation, followed by the bundling and subsequent breakdown of actin. Actin depolymerization using Lat B caused no change in YVADase activity. *In vivo* inhibition of YVADase activity prevented PCD and actin breakdown, therefore substantiating actin as a likely substrate for caspase-like proteases (CLPs). The mitochondrial pore inhibitor CsA significantly decreased YVADase activity, and prevented both PCD and actin breakdown; therefore suggesting the mitochondria as a possible trigger for CLPs during PCD in the lace plant. To our knowledge, this is the first *in vivo* study using either caspase-1 inhibitor (Ac-YVAD-CMK) or CsA, following which the actin cytoskeleton was examined. Overall, our findings suggest the mitochondria as a possible upstream activator of YVADase activity and implicate these proteases as potential initiators of actin breakdown during perforation formation via PCD in the lace plant.

## Introduction

### Programmed Cell Death (PCD) in Plants

Programmed cell death (PCD) is an active process resulting in the death of cells within an organism and is pervasive throughout eukaryotes. Within plant systems, developmentally regulated PCD occurs throughout the life cycle [1],[2]. Examples of developmentally regulated PCD include the deletion of the embryonic suspensor [1],[3] self-incompatibility (SI) in pollen [1],[2],[4] and leaf morphogenesis, as is seen in the lace plant (*Aponogeton madagascariensis*) [5]–[7]. The mitochondria [8],[9], caspases [10],[11] (*C*ysteine *ASP*artate-specific prote*ASES),* and the actin cytoskeleton [12] have been implicated in animal PCD, though less is known regarding the dynamics of these cellular components during PCD in plants.

### The Mitochondria and PCD

In Metazoans, mitochondria aid in PCD via the release of intermembrane space (IMS) proteins, including cytochrome *c* (cyt-*c*), into the cytosol [8],[9]. IMS proteins are often released via rupture or permeabilization of the outer mitochondrial membrane, usually as a consequence of mitochondrial permeability transition pore formation [9],[11],[13]. Mitochondria have also been implicated in plant PCD [2],[14]–[19] where the release of IMS proteins, including cyt-*c,* has been detected in a number of systems [15],[20]–[23]. However, work completed by Balk and Leaver (2003) [23] suggests that cyt-*c* release in plant systems may not be responsible for PCD activation. The mitochondrial permeability transition pore inhibitor cyclosporine A (CsA) has been shown to prevent PCD in a variety of animal [11],[24] and plant [18],[20],[25] systems.

Following the release of cyt-*c* in animal cells, it binds with apoptosis activating factor-1 in the cytosol forming the apoptosome, a complex that is capable of activating caspases to further PCD. Work completed by van der Biezen and Jones (1998) [26] suggested the role of an apoptosome-like structure in plants, one which is hypothesized to activate downstream caspase-like proteases (CLPs) [26]. Although additional research on the topic is scarce, it is suggested that plant resistance gene (R-gene) products may act analogously as controlling adaptors in a plant protein structure [27].

### Caspases and Caspase-like Proteases (CLPs)

Caspases play a fundamental role during PCD in animals, cleaving protein substrates adjacent to aspartate (Asp) residues [10],[11],[27]–[28]. Caspase-1 possesses a substrate specificity for the peptide sequence Tyr-Val-Ala-Asp (YVAD), while caspase-3 has been shown to have an affinity for Asp-Glu-Val-Asp (DEVD) [29]. Currently, no true caspases have been found within plant systems, although evidence suggests that CLPs exist [29]–[31]. A main group of CLPs, cysteine endopeptidases, are further subdivided into two groups: vacuolar processing enzymes (VPEs) [28],[30],[32] and metacaspases [28],[30],[33]–[34]. VPEs resemble true caspases in protein structure and recognize Asp when it is part of a YVAD sequence, analogous to caspase-1 [28],[32]. Synthetic caspase inhibitors have been used to stop PCD in both plant [30] and animal [10] systems and provide further evidence for CLPs in plants.

### The Cytoskeleton

Globular actin (G-actin) monomers assemble to form single strand filamentous actin (F-actin), two of these F-actin filaments are then wound together to produce a single microfilament, the main component of the actin cytoskeleton. Actin has been implicated in the plant PCD process [35], [36] where it undergoes a variety of morphological changes including: depolymerization followed by subsequent aggregation into punctate foci during SI in both *Papaver rhoeas* (poppy) [37] and *Pyrus pyrifolia* pollen [38] as well as bundling into dense cables during the hypersensitive response (HR) in tobacco BY2 cells [39].

The actin cytoskeleton has been shown to be an effector as well as a target during PCD signalling [40], [41]. In animals, caspases have been shown to cleave cytoplasmic proteins including actin [12],[27],[38],[39]. Conversely, in plants these associations are not as easily discernible. In 2006, Thomas et al. [42] concluded that actin depolymerization using Latrunculin B (Lat B) induced caspase-3-like activity in *Papaver rhoeas* pollen. This pathway of induction was confirmed in 2008 by Franklin-Tong and Gourlay, [12] where it was demonstrated that actin depolymerization using Lat B was sufficient to trigger CLP activity. However, in 2007 Vercammen et al. [43] proposed that the metacaspase gene *mcII-Pa* may, like animal caspases, regulate actin organization during *Picea abies* suspensor differentiation.

### The Lace Plant and Programmed Cell Death (PCD)

The lace plant (*Aponogeton madagascariensis*) is an aquatic monocot that forms perforations between longitudinal and transverse veins in spaces known as areoles, over its entire leaf surface via PCD [6],[7],[42]. PCD begins in the center of these areoles and radiates outwards, stopping four to five cells from the veins. The lace plant provides an ideal system for studying PCD for several reasons including: a developed method for sterile culture, nearly transparent leaves, as well as a predictable pattern of perforation formation [5],[6],[44]–[45]. The process of perforation formation has been previously divided into the five following stages: 1. Pre-perforation, 2. Window, 3. Perforation formation, 4. Perforation expansion, 5. Mature perforation [6]. Within a single areole of a window stage leaf, cell death has been further subdivided based on the progression of PCD, and is visibly discernable by colour differences [19]. The cells closest to the vasculature are control cells or non-PCD (NPCD); these cells are initially pink in colour due to the pigment anthocyanin found in the vacuole. The next division contains cells in the early stages of PCD (EPCD); green pigmentation is notable in these cells due to the abundance of chloroplasts and loss of anthocyanin. The third area is comprised of cells in the late stages of PCD (LPCD), which are nearly void of both pigments. Using this cell death gradient, the dynamics of organelles including the mitochondria, chloroplasts, actin cytoskeleton, nucleus and vacuole have been investigated throughout PCD. These observations were used to establish the order of cellular events during lace plant PCD [46].

This paper attempts to shed light on the interactions among the mitochondria, CLPs and the actin cytoskeleton during developmentally regulated PCD in the novel model species *A. madagascariensis*; biochemical analyses, pharmacological experimentation and advanced microscopy techniques were used to achieve this aim.

## Materials and Methods

### Plant Materials

Lace plants used for experimental purposes were grown in axenic cultures in magenta boxes and subcultured as described by Gunawardena et al. (2006) [46]. Plants were grown at 23.5°C with 12 h light (125 µmol·m^−2^·s^−1^)/12 h dark provided by daylight stimulating fluorescent bulbs (Phillips, Daylight Deluxe, F40T12/DX, Markham, Ontario). All chemicals were purchased from Sigma Aldrich (St. Louis, MO, USA), unless otherwise stated.

### Light Microscopy

Micrographs of leaf stages and representative half areoles following treatments were acquired utilizing differential interference contrast (DIC) optics and a digital camera (Nikon DXM 1200c) on an eclipse 90*i* compound microscope equipped with NIS elements acquisition and analysis software (Nikon, Mississauga, Ontario, Canada). All composite plates were assembled using Adobe Photoshop version 10.0 (Adobe Systems Inc., San Jose, California, USA).

### Confocal Laser Scanning Microscopy

Confocal investigations were performed utilizing an Eclipse T*i* microscope (Nikon, Mississauga, Ontario, Canada) with EZ-C1 3.80 imaging and analysis software. Fluorescent images were acquired via fluorescein isothiocyanate (excitation 460–500 nm, emission 510–560 nm) or tetramethylrhodamine isothiocyanate (excitation 527–552 nm, emission 577–632 nm).

### Pharmacological Treatments

#### Caspase-1 inhibitor

Caspase-1 inhibitor II (Ac-YVAD-CMK; Cat # 400012, Calbiochem, Darmstadt, Germany) is a cell permeable, irreversible L-1β Converting Enzyme (ICE) inhibitor. Five mg of the inhibitor was dissolved in 108.2 µl DMSO (final concentration 92.42 mM) and added directly into 20 ml sterile Murashige and Skoog (MS) medium (final concentration 0.462 mM), the solution was then filter sterilized. Healthy plants approximately 4 weeks of age with at least two perforated leaves were divided at random into experimental or control groups. The plants were then transferred aseptically into 40 ml glass vials (one per vial), into which the sterile caspase inhibitor+MS solution was added. Plants were allowed to grow for 7 days at which time the leaf that was produced was excised, photographed and stained with Alexa Fluor 488 phalloidin as described below.

#### Latrunculin B

A gradient of concentrations and incubation times for Lat B treatments were tested. Concentrations of 1 µM, 5 µM, 10 µM, 25 µM and 75 µM were tested at 3 and 6 hrs incubation, following which 25 µM and 75 µM were concluded to be toxic. Subsequently, 1 µM, 5 µM and 10 µM were tested at 10 min, 30 min, and 1 hour incubation. The combination of 1 µM in dH_2_O for 30 minutes was determined to be suitable. Window stage leaves were excised from whole plants and were treated with a Lat B solution (stock dissolved in dimethyl sulfoxide DMSO) at the time and concentration noted above in 20 ml petri dishes; control leaves received an equal amount of DMSO. Leaves were then rinsed thoroughly with dH_2_O and either stained with Alexa Fluor 488 phalloidin to examine the cytoskeleton,or pre-perforation leaves were assayed for CLP activity using the flourometric assay as described below. To ensure growth, development and experimental outcomes were accurate from excised leaf samples, whole plant experiments were also completed with Lat B. These experiments were completed in vials as described for caspase-1 inhibitor treatments above. No observable differences in parameters were noted between the excised leaf and whole plant Lat B experiments (data not shown).

#### CsA

Healthy plants approximately 4 weeks of age, containing at least 2 perforated leaves, were divided at random into experimental or control groups. Under aseptic conditions, liquid medium was poured out of each magenta box and replaced with 200 mL of fresh liquid medium; treatment groups received CsA stock solution (dissolved in 90% ethanol) to a final concentration of 10 µM (as optimized in Lord et al. 2011 [19]) and control plants received an equal volume of ethanol. Plants were then returned to growth racks under normal light conditions for 7 days until the first representative window stage leaf had formed, at which time the leaf was harvested, photographed and stained with Alexa Fluor 488 phalloidin as described below.

### In vitro Caspase Substrate Cleavage Assay

Lace plant leaves taken from varying stages of perforation formation (pre-perforation, window and mature; 6 independent experiments, 2 replicates per experiment), had their midribs removed, weighed, and then frozen overnight in liquid nitrogen. CsA or Lat B treated leaves (3 independent experiments, 2 replicates per experiment) used for the assay were at the pre- perforation stage of leaf development for comparison with non-treated samples. Samples were then ground in assay buffer (100 mM HEPES, 10% sucrose, 0.1% CHAPS, 5 mM DTT, pH 6.5) on ice. The tissue homogenate was centrifuged at 15,000 rpm at 4°C for 15 min and the supernatant was collected. Protein concentration in the cell extract was determined by Bradford assay (BioRad, Hercules, CA, USA). Proteolytic activity was measured in 160 µl reaction solution containing approximately 200 µg of protein and 50 µM of AFC-conjugated peptide (BioVision, Cat #K110-100, Milpitas, CA, USA) specific to mammalian caspase-1. Double reactions were incubated for 1.5 h at 37°C and fluorescence readings of ice-cold samples were taken at 5 min intervals with an excitation of 390/20 nm and an emission of 510/10 nm (Fluoroskan Ascent, Thermo Scientific, Marietta, OH, USA). Readings were measured and compared against a blank containing assay buffer and AFC-conjugated peptide, without protein. Kinetics of substrate hydrolysis were linear throughout the first hour of the reaction, following which the curve plateaued. A 0.005–0.5 µM AFC standard, diluted in assay buffer, was used to conclude the amount of fluorochrome released. Final proteolytic activity is expressed in pmol/min/mg protein cleaved. Active caspase-1 (BioVision, Cat.# 1081-25) was used as a positive control (data not shown). Each CsA and Lat B experiment contained two controls. The first being an experimental control, in which leaves were treated with the same solvent as the drug in use; a second control consisted of non-treated pre-perforation leaves where no solvent was used.

### Alexa Fluor 488 Phalloidin and Propidium Iodide Staining

Leaves were obtained from sterile cultures no more than 5 h prior to fixation. The staining protocol was modified from Wertman et al. [46] and Poulter et al. (2008) [47]. Initially, 10 mL of a 4% paraformaldehyde (BioShop Canada Inc., Burlington, Ontario, Canada) solution was made in an actin-stabilizing buffer (ASB; 100 mM Pipes (pH 6.80), 1 mM MgCl_2_, 1 mM CaCl_2_, 75 mM KCl) and set aside. Next, sterile leaves were cut into 5 mm by 5 mm pieces with a razor blade and placed in 10 mL of a 0.4 mM 3-Maleimidobenzoic acid *N*-hydroxysuccinimide ester (MBS) in ASB, for 10 minutes. After 10 minutes, the paraformaldehyde solution was added drop wise to the MBS solution for a final paraformaldehyde concentration of 2% and was incubated for 3 h at 4°C. Following fixation leaves were rinsed three times over 20 minutes in ASB and placed on a multi-welled slide. For leaf samples from all stages excluding mature, a staining solution containing 0.1 µM Alexa Fluor 488 phalloidin (Invitrogen Canada Inc., Burlington, Ontario, Canada) and 0.1% Triton X-100 in ASB was used. For mature stage leaf samples, a staining solution containing 0.2 µM Alexa Fluor 488 phalloidin (Invitrogen) and 0.1% Triton X-100 in ASB was used. Samples were placed in a humidity chamber, wrapped in tinfoil and incubated overnight at 4°C. Following overnight incubation, staining solution was aspirated off, samples were rinsed in ASB and subsequently stained with 0.5 mg/mL propidium iodide (PI) for approximately 5 minutes. Samples were again rinsed in ASB and mounted in Gel/Mount^tm^ (Biomeda Corp., Foster City, California, USA) on glass slides, cover slipped, sealed with clear nail polish, and viewed via confocal microscopy as described above.

### Actin width and Intensity Quantification

Both actin width and intensity were quantified given that the parameter width (measured within a single micrograph of a z-stack series) took into account the thickness of actin and alone was not a strong indicator of actin breakdown. This was evident from actin staining in LPCD cells that often retained actin thickness, but had been dramatically degraded at both ends. Intensity (measured in maximum projected z-stack micrographs) was able to take into account the degradation of actin microfilament bundles from the ends.

Actin width was measured by acquiring micrographs with the confocal microscope as described above. For non-treated control leaves approximately 50 images (approximately 25 z-stacks per image) were acquired for each stage of PCD. Approximately 150 filaments (3 filaments per image) for each stage were measured (5 independent experiments were completed, approximately 3 leaves utilized per experiment). For Lat B treated leaves approximately 20 images (25 z-stacks per image) were acquired for each stage of PCD. Approximately 50 individual filaments (approximately 2.5 filaments per image) for each stage were measured (5 independent experiments were completed, approximately 2 leaves utilized per experiment). For caspase-1 inhibitor treated leaves approximately 40 images (25 z-stacks per image) were acquired for each stage of PCD. Approximately160 individual filaments (4 filaments per image) for each stage were measured (3 independent experiments were completed, approximately 3 leaves utilized per experiment). For CsA treated leaves approximately 20 images (25 z-stacks per image) were acquired for each stage of PCD. Approximately 80 individual filaments (4 filaments per image) for each stage were measured (3 independent experiments were completed, approximately 3 leaves utilized per experiment). For more accurate width measurements, images from each stage of PCD, for each treatment were magnified using an after-capture zoom function on EZ-C1 3.80 analysis software. The mean of all widths from each treatment for each stage were then calculated for statistical analysis.

Actin intensity was measured by acquiring micrographs with the confocal microscope as described above. In all cases maximum projected micrographs were cropped to 1300 µm^3^ and intensity was measured in a.u. using EZ-C1 3.80 analysis software. For non-treated leaves approximately 40 images (40 z-stacks per maximum projected image) were acquired for each stage of PCD (5 independent experiments were completed, approximately 3 leaves utilized per experiment). For Lat B trials approximately 25 images (40 z-stacks per maximum projected image) were acquired for each stage of PCD (5 independent experiments were completed, approximately 3 leaves utilized per experiment). For caspase-1 inhibitor trials, approximately 20 images (40 z-stacks per maximum projected image) were acquired for each stage of PCD (3 independent experiments were completed, approximately 3 leaves utilized per experiment). Lastly, for CsA trials approximately 30 images (40 z-stacks per maximum projected image) were acquired for each stage of PCD (3 independent experiments were completed, approximately 3 leaves utilized per experiment). The means of all intensities for each treatment for each stage were then calculated for statistical analysis.

The data for width and intensity for each treatment (Lat B, caspase-1 inhibitor and CsA) from each stage of PCD (NPCD, EPCD and LPCD) was compared to two sets of control data; non-treated controls, and experimental controls, that were given an equal volume of the solvent used to dissolve the corresponding treatment.

### Statistical Analysis

Statistical analyses were carried out using a general linear model (GLM) analysis of variance (ANOVA) with Minitab 16 statistical software (Minitab Inc., State College, PA, USA). Individual means were compared using the Tukey test at 95% confidence intervals. Data are represented as mean ± S.E.M.

## Results

### Caspase-like Activity During PCD in the Lace Plant

**Figure 1:**
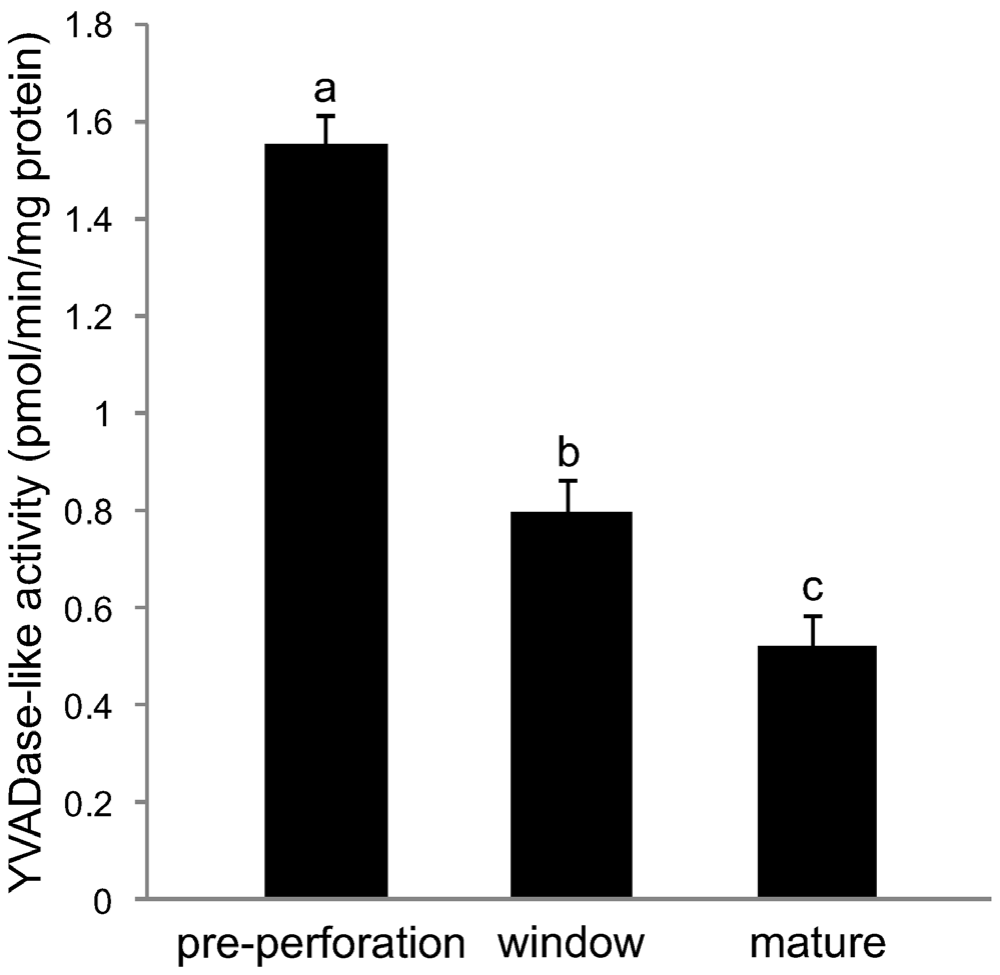
Kinetics of YVADase activity in non-treated control leaves. Leaf samples from pre perforation leaves (PCD not visibly detected), window stage leaves (PCD visibly occurring in central cells) and mature leaves (PCD complete) were used. Data are expressed as mean ± S.E.M. (*n* = 6). Data represented by different letters are significantly different (*P*≤0.05 ANOVA).

To determine if, and when CLP activity occurred during PCD in the lace plant, the ability of pre-perforation, window and mature stage leaves to cleave the synthetic peptide substrate YVAD-AFC was compared. The cleavage rates of the substrate varied significantly among all three stages of leaf development (*P*≤0.05; Figure 1).

### The Actin-cytoskeleton During PCD in the Lace Plant

**Figure 2:**
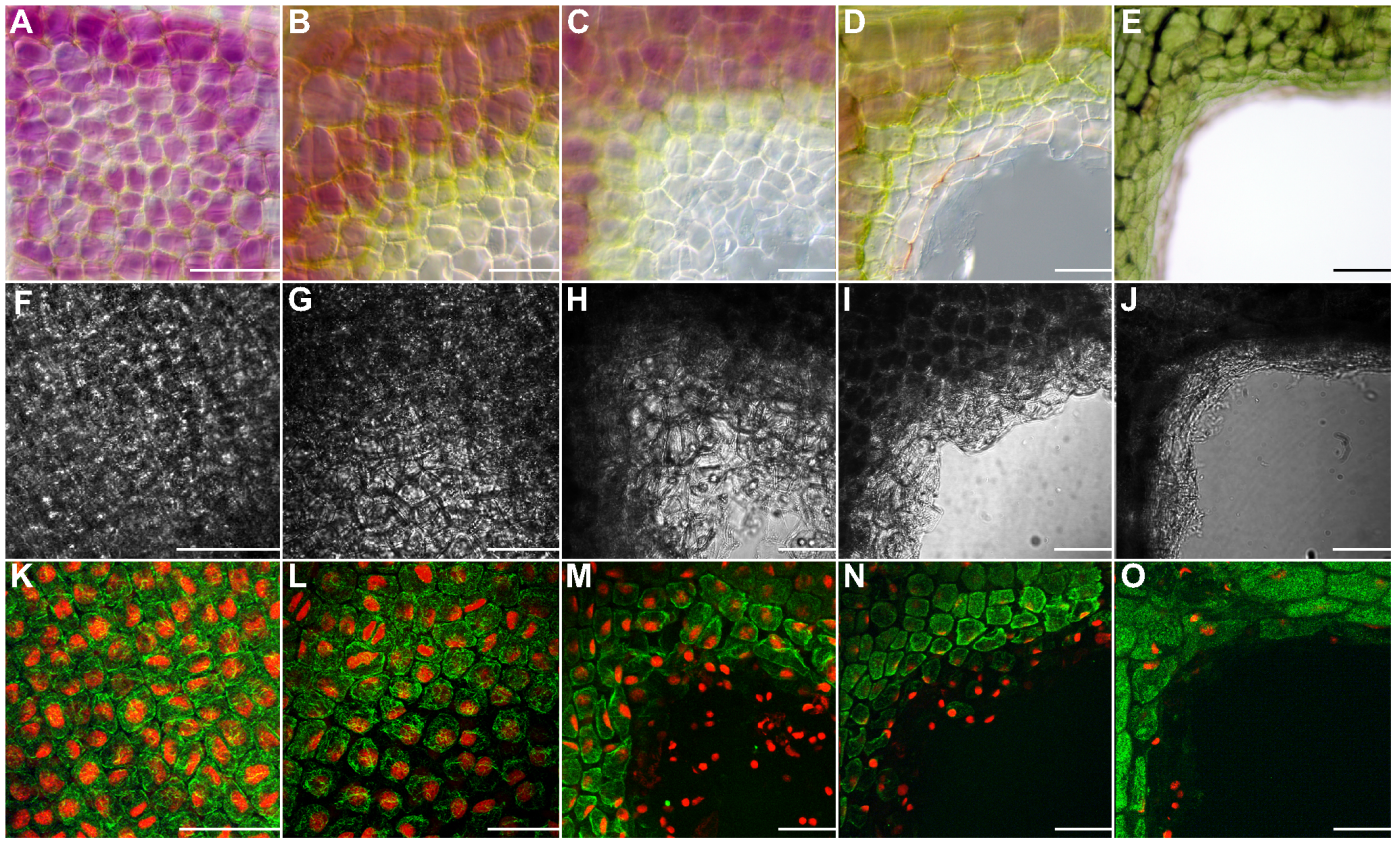
Rearrangement of the actin cytoskeleton during leaf morphogenesis over five stages of leaf development in the lace plant. Each image depicts a piece of a single corner of an areole (A–E). Coloured differential interference contrast (DIC) images of (A) pre perforation (B) window (C) perforation formation (D) perforation expansion and (E) mature stage leaf areoles. (F–J) Black and white DIC images of (F) pre perforation (G) window (H) perforation formation (I) perforation expansion and (J) mature stage leaves. (K–O) Fluorescent images of Alexa Fluor 488 phalloidin (green) stained areoles counterstained with propidium iodide (PI; red) of (K) pre perforation (L) window (M) perforation formation (N) perforation expansion and (O) mature stage leaves. Please note that images A-E do not correspond with fluorescent images F-O. These images are representative micrographs to illustrate the five stages of leaf development. DIC images F-J are corresponding to K-O. There is a consistent gradient of actin microfilament staining over the pre perforation areole; conversely there is variation (bundling followed by breakdown) in actin microfilament dynamics over the gradient of PCD (NPCD-LPCD) found within the single areole of the window stage leaf. Note the complete degradation and disappearance of actin microfilament staining as the perforation becomes larger, from perforation formation to the mature stage of leaf development. Scale bars = 70 µm.

Lace plant leaves were stained with Alexa Fluor 488 phalloidin to visualize actin and examined over the five stages of leaf development (Figure 2). In pre-perforation stage leaves actin appeared thin and organized, with no bundling or visual breakdown (Figure 2A, F and K). Conversely, in window stage leaves there appeared to be variations in actin organization across the gradient of PCD within an areole [46] (Figure 2B, G and L). Actin breakdown in the center of areoles was extensive in perforation formation leaves (Figure 2C, H and M) and continued to progress to the perforation border throughout leaf development, from perforation expansion (Figure 2D, I and N) to mature leaves (Figure 2E, J and O).

**Figure 3:**
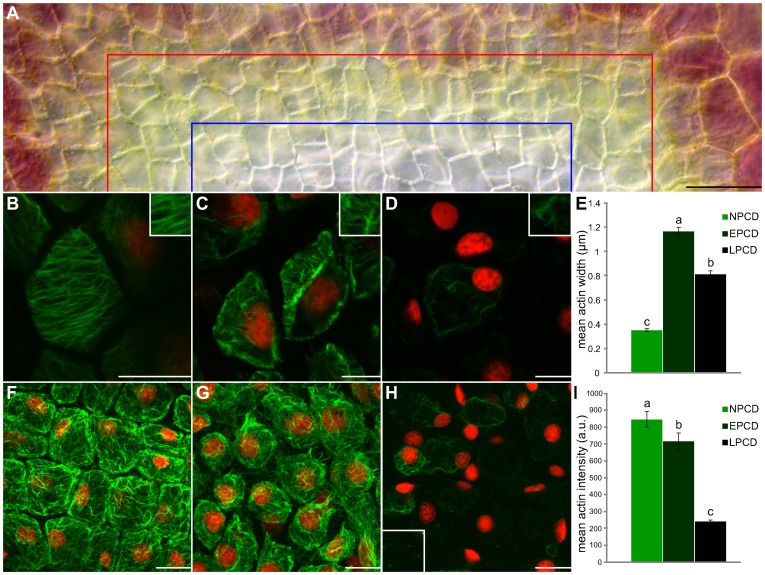
Rearrangement of the actin cytoskeleton over a gradient of PCD within a single areole of a non-treated window stage leaf. Cells within the lower portion of this figure (B–D and F-H) are stained with fluorescent Alexa Fluor 488 phalloidin (green) for actin and counterstained with propidium iodide (PI; red) for nuclei; tissues are fixed. (A) A representative DIC micrograph of half of a single areole of a window stage leaf demonstrating the gradient of PCD that exists over this region. Cells between the border of the image and the red line are NPCD cells and do not undergo PCD during perforation formation, cells between the red line and the blue line are in the early stages of PCD (EPCD) and cells between the blue line and the bottom of the image are in the late stages of PCD (LPCD). (B–D) Characteristic single z-stack micrographs of actin width within NPCD, EPCD and LPCD stage cells (0.38 µm;1.17 µm; 0.82 µm, respectively; see insets). (E) Mean widths of actin microfilaments. (F–H) Representative maximum projection micrographs of actin intensity within NPCD, EPCD and LPCD stage cells (848.72 a.u., 717.70 a.u. and 240.86 a.u, respectively). Note inset in panel H highlights punctate actin foci. All actin intensity measurements were acquired within 1300 µm^3^ of maximum projected z-stacks tissue. (I) Mean intensities of actin microfilaments. All error bars are representative of standard error and all data represented by different letters are significantly different within individual graphs (*P*≤0.05 ANOVA). Scale bars (A) = 50 µm, (B–D) = 15µm, (F–H) = 25 µm.

The gradient of PCD within a single areole of a window stage leaf was used to quantify actin dynamics during PCD on a detailed level (Figure 3). Both actin width and intensity were quantified. All mean actin widths varied significantly between NPCD (0.38 µm), EPCD (1.17 µm) and LPCD (0.82 µm; *P*≤0.05; Figure 3B–E). Groupings of actin within NPCD cells were thin, and often organized in a parallel fashion (Figure 3B; highlighted in inset). Actin organized into thick bundles during EPCD (Figure 3C; highlighted in inset) and began to breakdown during LPCD (Figure 3D; highlighted in inset). All mean actin intensities varied significantly among NPCD (848.72 a.u.), EPCD (717.70 a.u.) and LPCD (240.86 a.u.; *P*≤0.05; Figure 3F–I). NPCD cells illustrated a thin layer of actin that underlayed the PM in a fine mesh (Figure 3F). Actin within EPCD cells was less consistent and bundling of microfilaments caused gaps or breaks in the coating (Figure 3G). LPCD cells depicted actin that was severely broken down and often missing in particular cells. Less frequently, actin within LPCD cells was visualized as small punctate foci (Figure 3H; highlighted in inset).

**Figure 4:**
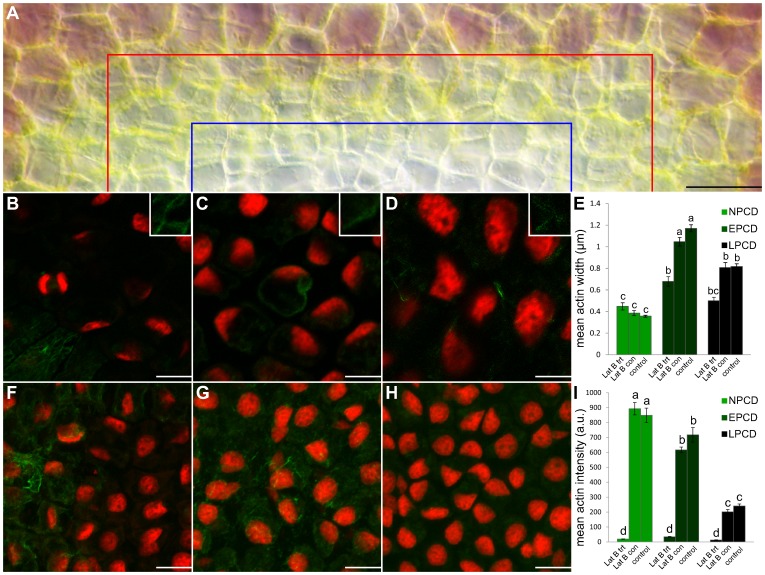
Actin depolymerization following treatment with Latrunculin B (Lat B). Cells within the lower portion of this Figure (B–D and F–H) are stained with fluorescent Alexa Fluor 488 phalloidin (green) for actin and counterstained with propidium iodide (PI; red) for nuclei; tissues are fixed (A) A representative DIC micrograph of a half of a single areole of a window stage leaf following Lat B treatment. Cells between the border of the image and the red line are representative NPCD cells, cells between the red line and the blue line are representative EPCD cells and cells between the blue line and the bottom of the image are representative LPCD cells. (B–D) Characteristic single z-stack micrographs of actin width within NPCD, EPCD and LPCD stage cells (0.44 µm, 0.68 µm, 0.5 µm, respectively; see insets) following Lat B treatment (E) Lat B experimental data compared to non-treated leaves (control; data extracted from Figure 3) and treated controls (Lat B con). (F–H) Representative maximum projection micrographs of actin intensity within NPCD, EPCD and LPCD stage cells (19.52 a.u., 34.35 a.u. and 13.0 a.u. respectively) following Lat B treatment. All actin intensity measurements were acquired within 1300 µm^3^ of maximum projected z-stacks tissue. (I) Mean actin intensities for each stage of PCD. Data for non-treated controls were extracted from Figure 3. All error bars are representative of standard error and all data represented by different letters are significantly different within individual graphs (*P*≤0.05 ANOVA). Scale bars (A) = 45 µm, (B–D) = 20 µm, (F–H) = 25 µm.

Following actin depolymerization with Lat B treatment, leaves were stained with Alexa Fluor 488 phalloidin and actin was quantified (Figure 4). Figure 4A displays half of a single representative areole following Lat B treatment; note that the areole appears as if it will produce a perforation as denoted by pigment loss in EPCD and LPCD stage cells. Both mean actin width and intensity were quantified. The mean actin width for NPCD cells (0.44 µm; Figure 4B and E) following Lat B treatment did not vary significantly from NPCD non-treated controls (0.38 µm; *P*≥0.05; Figure 4E). However, mean actin width for EPCD cells (0.68 µm; Figure 4C and E) and LPCD cells (0.50 µm; Figure 4D and E) following Lat B treatment did vary significantly from their non-treated controls (*P*≤0.05; Figure 4E). Examining mean actin intensities following Lat B treatment, NPCD (19.5 a.u.; Figure 4F and I), EPCD (34.35 a.u.; Figure 4G and I) and LPCD (13.0 a.u.; Figure 4H and I) all varied significantly from their non-treated controls (*P*≤0.05; Figure 4I). Lat B experimental controls did not vary significantly from non-treated controls for the same stage (*P*≥0.05; Figure 4I).

**Figure 5:**
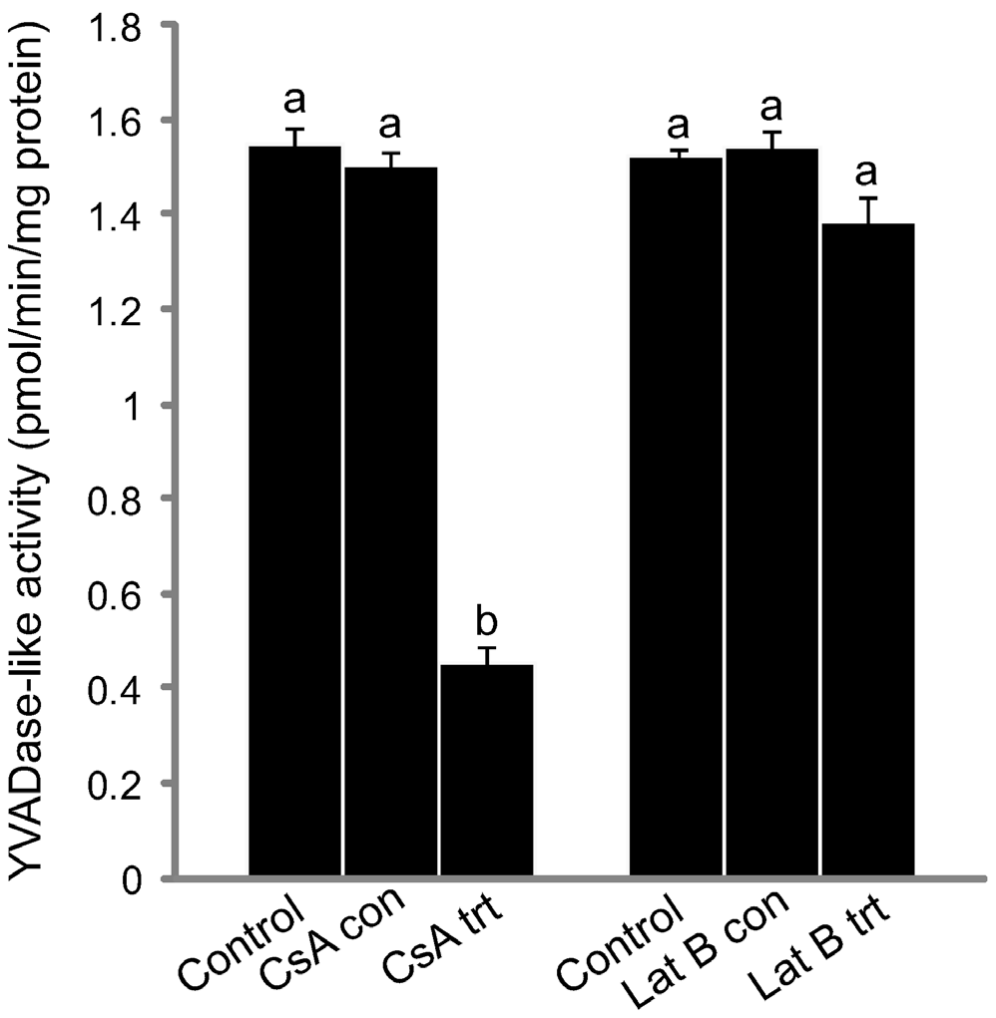
Kinetics of YVADase activity for cyclosporine A (CsA) and Latrunculin B (Lat B) treated leaves. CsA and Lat B pre-perforation stage leaf data as compared to their respective experimental and non-treated controls. Data are expressed as mean ± S.E.M. (*n* = 3). Data represented by different letters are significantly different (*P*≤0.05 ANOVA).

Additionally, pre-perforation leaves were treated with Lat B and CLP activity was measured using a caspase-1 fluorometric assay. We observed that cleavage rates of the synthetic peptide substrate (YVAD-AFC) did not diverge significantly from non-treated pre-perforation controls (*P*≥0.05; Figure 5). Mitochondria were also examined using Mito Tracker Red (CMXRos) following Lat B treatment and depicted normal mitochondrial staining (data not shown).

### Caspase-1 Inhibitors and Lace Plant PCD

**Figure 6:**
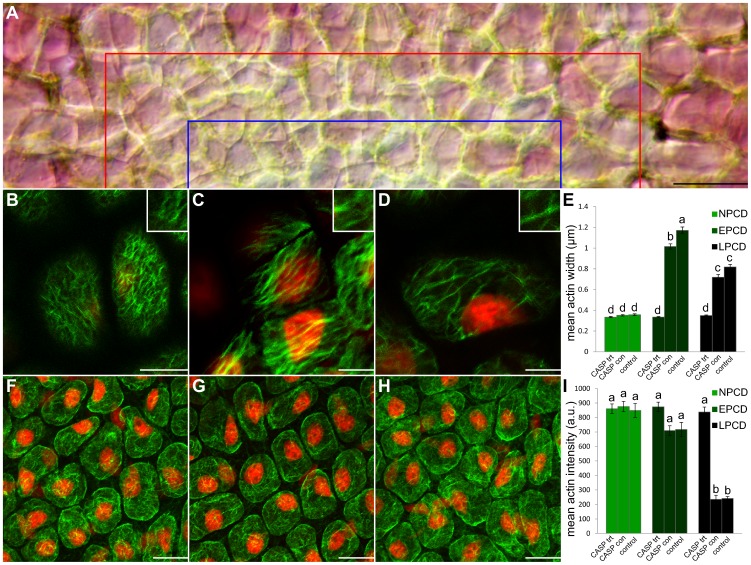
Actin dynamics following *in vivo* treatment with 0.462 M Caspase-1 inhibitor for 7 days in sterile culture. Cells within the lower portion of this figure (B–D and F–H) are stained with fluorescent Alexa Fluor 488 phalloidin (green) for actin and counterstained with propidium iodide (PI; red) for nuclei; tissues are fixed. (A) A representative DIC micrograph of a half of a single areole of a window stage leaf following caspase-1 inhibitor treatment; note that a loss of pigmentation is not present in this areole, indicating a perforation will not form. Cells between the border of the image and the red line are representative NPCD cells, cells between the red line and the blue line are representative EPCD cells and cells between the blue line and the bottom of the image are representative LPCD cells. (B–D) Characteristic single z-stack micrographs of actin width within NPCD, EPCD and LPCD stage cells (0.33 µm; 0.33 µm; 0.35 µm, respectively; see insets) following caspase-1 inhibitor treatment. (E) Caspase-1 inhibitor experimental data compared to non-treated leaves (control; data extracted from Figure 3) and treated controls (CASP con). (F–H) Representative maximum projection micrographs of actin intensity within NPCD, EPCD and LPCD stage cells (860.78 a.u., 874.10 a.u., 838.37 a.u. respectively) following caspase-1 inhibitor treatment. All actin intensity measurements were acquired within 1300 µm^3^ of maximum projected z-stacks tissue. (I) Mean actin intensities for each stage of PCD. Data for non-treated controls were extracted from Figure 3. All error bars are representative of standard error and all data represented by different letters are significantly different within individual graphs (*P*≤0.05 ANOVA). Scale bars (A) = 45 µm, (B–D) = 20 µm, (F–H) = 25 µm.

A caspase-1 inhibitor was applied *in vivo* and whole plants were then grown for 7 days. Leaves that emerged post-treatment contained no perforations; these leaves were subsequently stained with Alexa Fluor 488 phalloidin to observe actin (Figure 6). Figure 6A displays half of a representative areole following caspase-1 inhibitor treatment; note that a loss of pigmentation is not present in this areole, indicating a perforation will not form. Both mean actin width and intensity were quantified. The mean actin width for NPCD cells (0.33 µm; Figure 6B and E) following caspase-1 inhibitor treatment did not vary significantly from NPCD non-treated controls (*P*≥0.05; Figure 6E). However, mean actin width for EPCD cells (0.33 µm; Figure 6C and E) and LPCD cells (0.35 µm; Figure 6D and E) did vary significantly from non-treated controls (*P*≤0.05; Figure 6E). EPCD and LPCD mean actin widths did not vary significantly from NPCD non-treated controls (0.38 µm; *P*≥0.05; Figure 6E). Likewise, NPCD, EPCD and LPCD mean actin widths did not vary significantly from one another (*P*≤0.05; Figure 6E). Examining mean actin intensities following caspase-1 inhibitor treatment, NPCD (860.77 a.u.; Figure 6F and I) and EPCD (874.40 a.u.; Figure 6G and I) did not vary significantly from non-treated controls (*P*≥0.05; Figure 6I). However, LPCD (838.37 a.u.; Figure 6H and I) varied significantly from non-treated controls (*P*≤0.05; Figure 6I). EPCD and LPCD mean actin intensities did not vary significantly from NPCD non-treated controls (*P*≥0.05; Figure 6I). Likewise, NPCD, EPCD and LPCD mean actin intensities did not vary significantly from one another (*P*≤0.05; Figure 6I).

Additionally, all caspase-1 inhibitor treatment controls did not vary significantly from non-treated controls for the same stage (*P*≥0.05). Mitochondria were also examined via Mito Tracker Red CMXRos following caspase inhibition and depicted normal mitochondrial staining (data not shown).

### The Mitochondria, Actin and CLPs in the Lace Plant

**Figure 7:**
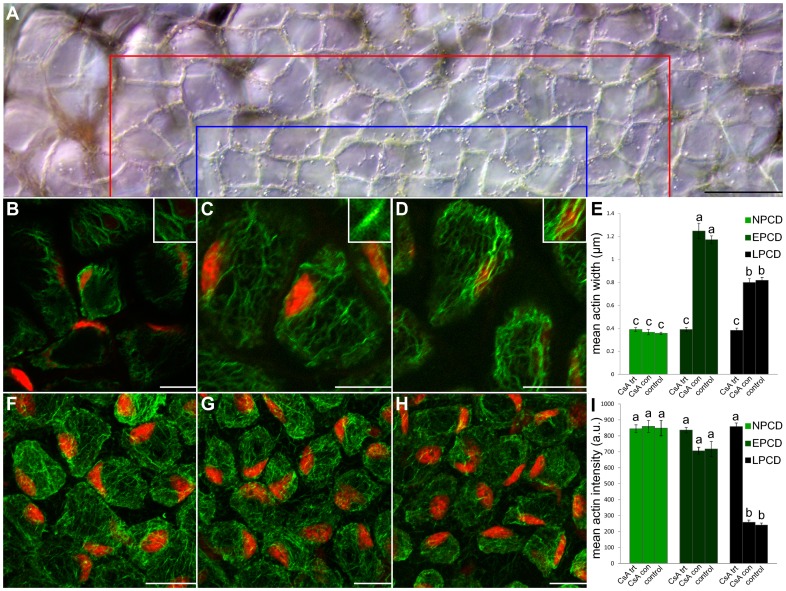
Actin dynamics following treatment with 10µM CsA for 3 days in sterile culture. Cells within the lower portion of this figure (B–D and F–H) are stained with fluorescent Alexa Fluor 488 phalloidin (green) for actin and counterstained with propidium iodide (PI; red) for nuclei; tissues are fixed (A) A representative DIC micrograph of a half of a single areole of a window stage leaf following CsA treatment; note that the typical gradient of cell death is not present indicating a perforation will not form. Cells between the border of the image and the red line are representative NPCD cells, cells between the red line and the blue line are representative EPCD cells and cells between the blue line and the bottom of the image are representative LPCD cells. (B–D) Characteristic single z-stack micrographs of actin width within NPCD, EPCD and LPCD stage cells (0.39 µm; 0.39 µm; 0.38 µm, respectively; see insets) following CsA treatment. (E) CsA experimental data compared to non-treated leaves (control; data extracted from Figure 3) and treated controls (CsA con). (F–H) Representative maximum projection micrographs of actin intensity within NPCD, EPCD and LPCD stage cells (844.50 a.u., 836.31 a.u., 858.30 a.u. respectively) following CsA treatment. All actin intensity measurements were acquired within 1300 µm^3^ of maximum projected z-stacks tissue. (I) Mean actin intensities for each stage of PCD. Data for non-treated controls was extracted from Figure 3. All error bars are representative of standard error and all data represented by different letters are significantly different within individual graphs (*P*≤0.05 ANOVA). Scale bars (A) = 45 µm, (B–D and F–H) = 25 µm.

To determine the role of the mitochondria in conjunction with CLPs and the actin cytoskeleton during lace plant PCD, plants were treated *in vivo* with CsA. Leaves that emerged following treatment contained no perforations; these leaves were subsequently stained with Alexa Fluor 488 phalloidin to quantify actin (Figure 7). Figure 7A displays half of a representative areole following CsA treatment. The typical gradient of cell death is not present indicating a perforation will not form (note: CsA treated leaves showed a different colouration from typical lace plant window stage leaves; perhaps due to the ethanol solvent). Both mean actin microfilament width and intensity were quantified. The mean actin width for NPCD cells (0.39µm; Figure 7B and E) following CsA treatment did not vary significantly from NPCD non-treated controls (*P*≥0.05; Figure 7E). However, mean actin width for EPCD cells (0.39µm; Figure 7C and E) and LPCD cells (0.38 µm; Figure 7D and E) did vary significantly from non-treated controls within their groups (*P*≤0.05; Figure 7E). EPCD and LPCD mean actin widths did not vary significantly from NPCD non-treated controls (*P*≥0.05; Figure 7E). Likewise, NPCD, EPCD and LPCD mean actin widths did not vary significantly from one another (*P*≤0.05; Figure 7E). Examining mean actin intensities following CsA treatment, NPCD (844.50 a.u.; Figure 7F and I) and EPCD (836.31 a.u.; Figure 7G and I) did not vary significantly from non-treated controls (*P*≥0.05; Figure 7I). However, LPCD (858.30 a.u.; Figure 7H and I) varied significantly from non-treated controls (*P*≤0.05; Figure 7I). EPCD and LPCD mean actin intensities did not vary significantly from NPCD non-treated controls (*P*≥0.05; Figure 7I). Likewise, NPCD, EPCD and LPCD mean actin widths did not vary significantly from one another (*P*≤0.05; Figure 7I). Additionally, all CsA treatment controls did not vary significantly from non-treated controls for the same stage (*P*≥0.05).

Following treatment of whole plants with CsA, leaves at the pre-perforation stage of development were assayed for CLP activity using a caspase-1 flourometric assay. Cleavage rates of the synthetic peptide substrate (YVAD-AFC) diverged significantly from non-treated control pre-perforation leaves (*P*≤0.05; Figure 5).

## Discussion

### Caspase-like Activity during PCD in the Lace Plant

Data presented here suggests that perforation formation within lace plant leaves requires the activation of a protease or group of proteases, which preferentially cleave YVAD sequence-containing substrates. Cleavage of a substrate containing YVAD mimics activity of caspase-1 during mammalian apoptosis [30],[45]. CLP (caspase-1) activity was determined to be the highest during the pre-perforation stage of leaf development, as compared to window and mature stage leaves (Figure 1). Thus it is likely that CLP activity is higher prior to the visible initiation of PCD as compared to mature stage leaves, where the process is complete. This hypothesis would be better concretized via CLP activity measurements in leaf primordia found surrounding the shoot apical meristem, prior to the pre-perforation stage of leaf development; however obtaining sufficient protein concentrations from these small leaf primorida is not feasible. This early initiation of CLP activity was also noted during Norway spruce autophagic cell death in embryonal tube cells where *in vivo* studies identified VEIDase (caspase-3) activity at the beginning of the execution phase of cell death [36].

### The Actin-cytoskeleton During PCD in the Lace Plant

The actin cytoskeleton has been implicated in plant PCD [35] and therefore was examined extensively in the present study. Across the five stages of leaf development it was apparent that visible alterations in actin did not begin to occur until the window stage of leaf development (Figure 2); this evidence indicates that CLPs (highest during the pre-perforation stage), are likely upstream of actin microfilament modifications. The thin linear actin seen in NPCD (Figure 3B) is essential as a framework for cellular structure, and allows myosin-based motors to transport organelles throughout the cytosol [38]. The bundling of actin seen in EPCD (Figure 3C) is comparable to the longitudinal thick fibres found during embryogenesis in *Picea abies,* [48]–[50] or the bundles of actin directed towards infection sites following the HR in *Arabidopsis* [51]. The reason for this bundling within the lace plant system is still not well understood and requires further investigation. The breakdown of actin, followed by its subsequent aggregation into punctate foci seen in LPCD (Figure 3H, highlighted in inset) is reminiscent of actin dynamics during SI in both *Papaver rhoeas* [37] and *Pyrus pyrifolia* pollen [38]. However, in *Papaver rhoeas* this depolymerization was necessary and sufficient for triggering PCD [12],[40].

The application of the actin depolymerization drug Lat B (1 µM) showed normal leaf growth and PCD processes (data not shown). Therefore, it can be concluded that actin microfilaments may not be crucial executors of PCD as described in poppy pollen, [12],[40] but may be downstream substrates for CLPs. Figure 4 displays cytoskeleton dynamics following Lat B treatment. Actin width and intensity varied significantly between treatments and non-treated controls at every point except for NPCD actin width. This reduction in the breakdown of filamentous actin in NPCD cells suggests a limited effect of the depolymerization agent, and infers the presence of very stable microfilaments, perhaps as a consequence of these cells not being genetically primed to die. No punctate foci were present within Lat B treated samples, signifying that foci produced during lace plant PCD may not be as resistant as those found in *Papaver rhoeas* [37], where it was reported that foci were extremely stable and unaffected by treatment with actin depolymerizers. Within metazoan PCD, it has been demonstrated that caspases act upon the actin cytoskeleton. In the lace plant system, actin was depolymerized via Lat B within pre-perforation leaves and CLP activity measured. Results suggest that cytoskeleton depolymerization (1 µM for 30 min) has no effect on CLP activity (Figure 5) thus providing additional evidence for CLPs upstream of actin cytoskeleton breakdown. As noted above this sequence of events is in contrast to the pathway recognized in *Papaver rhoeas,* where actin is thought to be the trigger of CLP activity [12],[38],[40]; however our results are in agreement with evidence from animal systems where caspases cleave filamentous actin [12],[27],[38],[39]. The authors speculate that the use of a higher concentration of Lat B may be able to induce CLP activity, possibly through a non-developmental PCD pathway, although this requires further investigation.

### Caspase-1 Inhibitor and the Lace Plant

To determine if CLPs were acting upon the actin cytoskeleton, causing breakdown, as is seen within metazoan PCD, caspase-1 inhibitor (Acetyl-YVAD-aldehyde or Ac-YVAD-CMK) experiments were completed. To the best of these authors knowledge, this is the first example in which a whole plant has been treated *in vivo* with such an inhibitor. Ac-YVAD-CMK is a commonly used inhibitor within plant systems with, Ac-YVAD-CHO shown to suppress cell death in tobacco mosaic virus (TMV) induced PCD, while Ac-DEVD-CHO had no effect [52]. On the contrary, during PCD in *Papaver rhoeas* pollen SI, Ac-DEVD-CHO was shown to suppress PCD while Ac-YVAD-CHO did not [4],[52],[53]. Additionally, for the first time within the literature, actin width and intensity were quantified following CLP inhibition. Results depicted that all NPCD, EPCD and LPCD treatments did not vary significantly from NPCD non-treated controls (Figure 6); this suggests that the inhibition of YVADase activity blocked actin dynamic changes. Overall, these *in vivo* experiments further support the notion that YVADase activity may play a role in actin breakdown during leaf morphogenesis in the lace plant.

### The Mitochondria, Actin and CLPs in the Lace Plant

Within metazoan PCD it has been demonstrated that the release of cyt-*c* and other IMS proteins from the mitochondria have the ability to activate caspases. CsA has been shown to prevent perforation formation in lace plant leaves [19], and therefore it was used in this study within pre-perforation leaves (where CLP activity was originally determined to be the highest) following which CLP activity was measured. Subsequent to CsA application, YVADase activity was determined to be significantly lower than controls (Figure 5), indicating that the mitochondria act upstream of YVADase activation. Following CsA treatment actin dynamics were also quantified (Figure 7). Lower CLP activation via the mitochondria presumably meant fewer CLPs affecting the actin cytoskeleton; this hypothesis was supported by results indicating that the actin cytoskeleton of CsA treated samples did not differ significantly from NPCD controls, or among stages (Figure 7). It should be noted however that although cyt-*c* release has been described in a variety of plant species, that this release does not appear to be the sole trigger for PCD in plants [23]. Therefore further investigation is required in order to understand how CsA prevents PCD in the lace plant.
